# From ‘Virgin Births’ to ‘Octomom’: Representations of Single Motherhood via Sperm Donation in the UK News

**DOI:** 10.1002/casp.2288

**Published:** 2016-11-03

**Authors:** S. Zadeh, J. Foster

**Affiliations:** ^1^ Centre for Family ResearchUniversity of CambridgeCambridgeUK; ^2^ Department of PsychologyUniversity of CambridgeCambridgeUK

**Keywords:** single motherhood, sperm donation, social representations theory, media representations, biotechnology

## Abstract

The use of sperm donation by single women has provoked public, professional and political debate. Newspapers serve as a critical means of both broadcasting this debate and effecting a representation of this user group within the public sphere. This study uses the theory of social representations to examine how single motherhood by sperm donation has been represented in the UK news over time. The study sampled news coverage on this topic in eight British newspapers during three 4‐year periods between the years 1988 and 2012. The dataset of news reports (*n* = 406) was analysed using a qualitative approach. Findings indicated that UK media reports of single women using donor sperm are underpinned by conventional categories of the ‘personal’, the ‘traditional’ and the ‘natural’ that when paired with their corollaries produce a representation of this user group as the social ‘other’. The amount of coverage on this topic over time was found to vary according to the political orientation of different media sources. Using key concepts from social representations theory, this article discusses the relationship between themata and anchoring in the maintenance of representations of the social ‘other’ in mass mediated communication. Findings are explained in relation to theoretical conceptions of the mass media and its position within the public sphere. It is argued that the use of personal narratives in news reports of single mothers by sperm donation may have significant implications for public understandings of this social group. © 2016 The Authors. *Journal of Community & Applied Social Psychology* published by John Wiley & Sons Ltd.

## Introduction

Arguments against single women using donor sperm have pervaded debates about who ought to be permitted access to assisted reproductive technologies (ARTs) in the UK since the 1940s (Barton, 1943). More recently met with disapproval from politicians (McCandless & Sheldon, 2010), fertility professionals (Lee, Macvarish, & Sheldon, 2012) and the general public (Kailasam, Sykes, & Jenkins, 2001), it is clear that despite changes to prior emphases on children's ‘need for a father’ in the legislation on assisted reproduction (Human Fertilisation and Embryology Act, 2008), single women using donor sperm remain at the heart of concerns about the choice to have a child, the meaning of motherhood, and the future of family life. This article seeks to contribute to understanding the social responses to single mothers by sperm donation by scrutinising UK media representations of this social group through the lens of social representations theory.

The theory of social representations (SRT) offers a clear framework for thinking about the ways in which social actors create, develop and share knowledge, particularly when faced with new or unfamiliar phenomena (Moscovici, 1984). SRT regards representations as the outcome of the relationship between the self, social others, and a specific social phenomenon, also termed the Ego‐Alter‐Object relation (Moscovici, 1972). According to the theory, it is the self's capacity to think about the social world in terms of others, and to construct reality *in relation to* those others, that forms the basis of social knowledge. As SRT scholars understand it, the relation between Ego and Alter in communication gives rise to representations, and representations are equally the means by which Ego and Alter can communicate.

In terms of the structure of representations, it has been theorised that representations are organised around a ‘figurative kernel’ or ‘thema’ (Moscovici, 2011) that is the result of thinking in oppositions, something taken to be true of all social thought (Billig, 1987). Such oppositional thinking is said to generate themata (and hence social representations) only in particular social and historical contexts (Marková, 2003) in which tension between antinomies arises (Moloney, Gamble, Hayman, & Smith, 2015); examples include left/right, in the social representation of political parties (Moscovici, 2011); morality/immorality, in the social representation of AIDS (Joffe, 1995); and certainty/uncertainty, in the social representation of global warming (Washer & Joffe, 2006). In other instances, oppositions in thinking may remain dormant, that is to say passed from generation to generation without reflection, although it is clear that all oppositions *have the potential* to become thematised. Conversely, themata are evidenced by their symbolic role in communication, that is, in the genesis and maintenance of representations. Beyond structuring representations, some themata have been determined ‘essential to the survival and enhancement of humanity’ (Marková, 2003:188), while others have been said to precede, and determine the nature of, majority/minority relations (Moscovici, 2011). The so‐called ‘basic themata’ are theorised as responding to ‘basic needs’ and ‘social drives’, such as the desire for social recognition, and may be implicated in the generation of many social representations, including those that seem to correspond to disparate phenomena (Marková, 2003).

The task of social representing is widely understood to be underpinned by two sociocognitive processes ‐ objectification and anchoring ‐ that involve naming a new phenomenon, and situating it in existing structures of meaning (Moscovici, 1984). It has been argued that these processes of meaning‐making are particularly marked in times of scientific or technological innovation (Moscovici, 1998), and may lead to the production of different representations by different social groups (Bauer & Gaskell, 2008). A more recent addition to the theory has grappled with the idea of ‘emotional anchoring’, by which new phenomena are attached to positive or negative emotions, as in fear or hope (Höijer, 2010). Relatedly, it has been suggested that media reports of biotechnological innovation may ignite a process of ‘collective symbolic coping’ (Wagner, Kronberger, & Seifert, 2002) through which phenomena that pose a threat to particular social groups are rendered intelligible and ultimately normalised. SRT scholars focussing on ARTs specifically have emphasised how new technologies are anchored in existing representations of nuclear family life by both professionals and the general public, with each of these groups expressing concerns in relation to the significance of genetic connections (Walker, Broderick, & Correia, 2007). The only SRT study to have explored media representations of ARTs examined 180 reader letters to two national Australian newspapers, providing insight into the views of the general public following legislative changes that now enable the use of ARTs by single women and female same‐sex couples in Australia (Correia & Broderick, 2009).

SRT is particularly well‐suited to analyses of the mass media because of its emphasis on the central role of communication in the generation of social knowledge. Within the theory, scholars have suggested that the critical question is not whether media communication impacts upon social representations, or vice versa, but what is acting upon both (Rouquette, 1996). It has relatedly been argued that the mass media is best understood as one dimension of the public sphere (Gaskell & Bauer, 2001) in which social representations are created, circulate and change over time (Bauer & Gaskell, 1999). The proliferation of mass mediated communication has been described as especially relevant to representations in modern culture (Jovchelovitch, 1997), characterised by ‘social representation makers’ (Moscovici, 1987) who depend in part upon the media for their genesis. It has moreover been argued that the ‘media shape the content, structure, form and speed with which representations spread in groups’ (Wagner, 1998:305) in ways unmatched by interpersonal communication alone. Particular attention has been paid to the ways in which mass mediated communication may be socially recognised – and legitimised – in ways that ‘local knowledges’ are not (Jovchelovitch, 1997). Put differently, such communication has been theorised to have at its unique disposal the resources for the production and proliferation of ideas (Wagner, 1998) in a public sphere of ‘unequally equipped agents’ (Jovchelovitch, 1997).

Regarding technological innovation, relationships between media reporting and reader responses to organ donation (Morgan, Movius, & Cody, 2009), genetically modified food (Bauer, 2002) and cloning (Väliverronen, 2004) have been established. Regarding ARTs, it has been argued that media reports may define and delimit public discussion and debate, and may be depended upon by those who otherwise have little or no communication about these phenomena (Franklin, 1990; Michelle, 2007). While very few scholars have adopted an SRT approach to the study of ARTs, the literature on mass media reports of these technologies is growing. In general, research has shown that reports either explicitly focus on opposite‐sex couples using ARTs, or have sought to either assimilate (Riggs, 2012) or repudiate (Alldred, 1998) the use of ARTs by same‐sex couples. Recent studies have also examined the role of fictional accounts in valorising ‘traditional’ parenting by opposite‐sex couples who are genetically related to their children (Shalev & Lemish, 2013). Single women specifically have been shown to be largely portrayed as a group who struggle with the practicalities of ‘Plan B’ (Silbergleid, 2002) such that they may abandon it all together; in both novels and films, stories of this user group often conclude with the instatement of the ‘traditional’ family (Maher, 2014).

Analyses of non‐fictional media about single women using donor sperm are, however, largely absent, with two studies having been conducted on this topic to date (Correia & Broderick, 2009; Michelle, 2006). The present study is the first to systematically examine mass media representations of single mothers by sperm donation in the UK news. In particular, it aims to address two of the questions identified by Rouquette (1996) as significant for SRT analyses of media communication: (i) what ‘rhetorical resources’ are used in relation to the structural properties of social representations? and (ii) how are the different components of social representations ‘treated’ by the media, both at a given moment, and over time?

## Method

Eight British newspapers ranging in political orientation (left‐leaning, right‐leaning and centrist) were chosen for analysis: the four most widely read broadsheets, ‘The Guardian’, ‘The Independent’, ‘The Times’ and ‘The Daily Telegraph’, and the four most widely read tabloids, ‘Daily Mirror’, ‘Daily Mail’, ‘The Sun’, and ‘Daily Express’ (Audit Bureau of Circulations, 2013). Sunday editions of these newspapers (‘The Observer’ and ‘The Mail on Sunday’) were also included. LexisNexis and Factiva were used to retrieve relevant articles over three 4‐year periods decided upon on the basis of political activity and inactivity about single women seeking donor sperm (1 January 1988 to 1 January 1992, 1 January 1998 to 1 January 2002, and 1 January 2008 to 1 January 2012). In 1990, the first UK legislation on ARTs – which stated that clinicians needed to consider a ‘child's need for a father’ in deciding whom to offer treatment – was implemented. In 2008, this clause was amended to a ‘child's need for supportive parenting’. In the intervening period (1998–2002), no such political changes took place, serving, therefore, as a useful point of comparison.

In order to generate the dataset, four combinations of search terms were initially used: (i) ‘unmarried’ and ‘donor’; (ii) ‘unmarried’ and ‘insemination’; (iii) ‘single’ and ‘donor’; and (iv) ‘single’ and ‘insemination’. This first stage yielded a total of 343 relevant newspaper articles. A second phase of data collection from a print archive of UK articles published on the subject of ARTs between the years 1990 and 1995 yielded a further 14 articles on the topic. Acknowledging data collection and analysis as an iterative process (Bauer & Aarts, 2000), a third phase involved the retrieval of electronic newspaper articles from each time period using the search terms (v) ‘virgin birth’ and (vi) ‘single mothers by choice’, identified as relevant from the first reading of all articles from each time period. The final dataset comprised 406 articles (Table 1).

**Table 1:** All newspaper articles from all data sources classified as right‐leaning (R), left‐leaning (L) or politically centrist (C) by date of publication

Newspaper	1988–1992	1998–2002	2008–2012
Daily Express and Sunday Express (R)	3	3	16
The Guardian and The Observer (L)	28	21	30
The Independent and The Independent on Sunday (C)	24	17	12
Daily Mail and The Mail on Sunday (R)	6	35	53
Daily Mirror and Sunday Mirror (L)	0	17	13
The Sun (R)	2	2	15
The Daily Telegraph and The Sunday Telegraph (R)	4	2	22
The Times and The Sunday Times (R)	28	19	34
Total	95	116	195

Data were analysed using Atlas.ti according to the principles of thematic analysis (Braun & Clarke, 2006). A total of 175 codes were created across all three subsets. Some of the codes appeared across subsets (e.g. ‘marriage’), whereas others did not (e.g. ‘British Pregnancy Advisory Service’). Some focussed on the devices employed within the article (e.g. ‘reference to/quote from Conservative MP’), and others were descriptive (e.g. ‘single motherhood as selfish’). Articles (and particular passages within each article) were often coded more than once, and the co‐occurrence of codes was considered at later stages of analysis. One month later, 10 articles from each subset were recoded, and no new codes were generated. Ongoing analyses in relation to the research questions resulted in the creation of 20 subthemes from the initial codes. These subthemes were reviewed, firstly according to data within each subset, and then according to the entire dataset. A thematic chart, depicting 3 main themes and 19 subthemes, was then developed (Table 2). The process of data collection and analysis was subject to theoretical and methodological scrutiny through a data audit conducted according to Flick's (2014) guidelines by an expert in research into families formed through the use of ARTs.

**Table 2:** Thematic chart depicting subthemes and their correspondence to three themata: (1) Personal/public, (2) Traditional/non‐traditional, (3) Natural/unnatural

*Age* (1,3)	*Motherhood*/*fatherhood* (2,3)
*Anonymous*/*identity‐release donation* (1,2)	*Personal narratives* (1,3)
*Biological clock* (3)	*Plan B* (2)
*Celebrity culture* (1,3)	*Political debate* (1)
*Children*'*s identity* (2,3)	*Popular culture* (3)
*Consumption* (3)	*Selfishness* (3)
*Female*/*male* (2,3)	*Sex*/*the syringe* (2,3)
*Genetic connections*/*missing links* (2)	*UK*/*overseas* (1,2)
*Government spending* (1)	*Welfare of child* (1,2)
*Health*/*disease* (1,2)	

## Results

The dataset was characterised by three main themes and their corollaries, expressed in terms of binary oppositions: personal/public, traditional/non‐traditional, and natural/unnatural. Analyses showed that the use of these themata in newspaper articles across all media sources in all time periods served to ostensibly normalise at the same time as ultimately portraying single women who use donor sperm as socially deviant. Differences were found in the number of articles published by different newspapers according to political orientation, with right‐leaning newspapers augmenting their coverage, and others either sustaining or decreasing their coverage over time (Table 1). No differences in thematic content were found according to political orientation, or between broadsheet and tabloid newspapers. Some differences emerged in terms of the emphases placed upon particular thematic aspects, and the language used to discuss them in either positive or negative terms. Discussion of the three themata, and the most prominent subthemes within them, will illustrate this.

### Personal/public

The issue of whether the use of donor sperm by single women was a personal decision or a public issue was present in both broadsheet and tabloid news at all three time points. In 1988–1992, articles focussed on the then‐occurring political debate about whether or not single women should be legally permitted to conceive through sperm donation. This debate was portrayed as between Labour and Liberal Democrat MPs on the one hand, and Conservative MPs on the other, with the latter depicted as favouring the prohibition of single women seeking fertility treatment.

*Lord McGregor of Durris* (*Lib Dem*) *asked how the prohibition would be enforced*. *Would women have to present themselves at clinics for treatment carrying their marriage lines*? *The clause would be regarded by married and unmarried people alike as a gross violation of the privacy of their intimate relations and lives*. ( The Times, 08.02.1990)

In keeping with one of the central tenets of SRT, that new phenomena incite a struggle over meaning between different social groups (Moscovici, 1988), articles in 1998–2002 were less likely to describe the use of donor sperm by single women as a political issue. Nevertheless, reports at this time continued to depict the cohort as the focus of public debate, and in 2008–2012, the presentation of single motherhood by sperm donation as a political concern resurfaced. Reports at this time focussed on proposed legislative changes to replace the clause requiring clinicians to consider a child's ‘need for a father’ with the ‘need for supportive parenting’.

*The Embryology Bill threw up an amendment legislating for a need for fathers*. *It*'*s a proposition many of us react to warmly*, *but for some particular reason we end up in a corner with small‐state*, *pro‐life*, *Eurosceptic*, *voucher‐fiends complaining about political correctness* (*it*'*s gone mad you know*)… *The other side*, *with equal and opposite vigour*, *scoffs at the nuclear family*, *supports comprehensive education*, *the multiplication of human rights*, *and says* ‘*unlicensed*, *unregulated sperm*’ *without facial revulsion*. ( The Independent, 21.05.2008)

In all three time periods, reports also raised the issue of whether or not women without partners ought to have their treatment financed by public health funds.

*Single women*, *women over 60*, *and lesbian and gay couples could all go to court to demand treatment on the NHS*. *Anyone could claim the* ‘*right*’ *to have children*. *Even convicted murderers could get in on the act*. ( Daily Mail, 30.03.2000)

Also apparent in research on media representations of older mothers (Campbell, 2011), the relationship between single motherhood and public funding was particularly prominent in 2008–2012. One article, for example, reported that ‘*women not in relationships are receiving publicly‐funded IVF despite official guidance*’ (The Daily Telegraph, 24.10.2011), and another juxtaposed them with ‘*others*’ more deserving of ‘*already stretched*’ services (Daily Mail, 19.03.2011). Other articles capitalised on this issue in headlines such as ‘*IVF for older women on the NHS*’ (Daily Mail, 28.06.2010), ‘*Single women being offered IVF on the NHS*’ (The Daily Telegraph, 24.10.2011) and ‘*Milking of the health service*’ (Daily Mail, 04.07.2011), and others still emphasised the more general costs associated with single motherhood. In particular, reports implied that such women would be unable to financially sustain themselves and their future children, even if they were able to meet the costs of treatment.

*It*'*s hard to keep up with the narrative*, *isn*'*t it*? *A sixtysomething retired shopkeeper with no husband*, *only a pension and state benefits for an income* … *chooses to be a mother*. ( The Daily Telegraph, 17.07.2009)

A number of articles in 2008–2012 focussed on singleton Nadya Suleman, the so‐called ‘Octomom’ who immediately became the object of media attention after giving birth to donor‐conceived octuplets. Several articles described how Suleman was unemployed and receiving disability benefits at the time of fertility treatment. Media reports generally portrayed Suleman as ‘excessively reproductive’ at the same time as she was deemed ‘excessively consumptive’ of public resources, echoing other representations of working‐class women in popular culture (Tyler & Bennett, 2010).

*But fascination with the so‐called* ‘*Octomom*’ *turned to almost universal revulsion when it was revealed that the 34‐year‐old is single*, *unemployed and already had six children under the age of eight*… *Even worse*, *she was claiming* £*2*,*500 a month from the state of California in food stamps and benefits*. ( The Mail on Sunday, 08.11.2009)

Alongside articles about Suleman's extraordinary experience of fertility treatment, several reports in all three time periods also included ‘ordinary’ mothers' narratives of their experiences. These narratives were increasingly employed in the news over time and most often depicted the relationship between womanhood and motherhood as inviolable.

*For as long as Tamsin Mitchell can remember*, *she has wanted a child*. *By her late thirties she had a successful career*, *was financially stable and was happy*. *There was just one missing ingredient*: *a man*. *She was also running out of time*. ( The Times, 11.07.2001)

Although the inclusion of personal narratives in news reporting on other topics has been identified as a means of inciting sympathy among readers (Foster, 2006), it was common for reports in 1998–2002 in particular to detail the so‐called ‘real‐life’ stories of single mothers by sperm donation alongside those of donor‐conceived adults, and specifically those who described their experience of finding out about their donor origins negatively.

“*I talk about donor insemination quite freely in front of him and when he*'*s old enough I will explain to him how he was conceived*. *I hope he understands that I did it out of love*.” … “*For years something made me suspect the man I was told was my father*, *Wilfred Bartlett*, *wasn*'*t my real dad* … *somehow I knew.*… *It was five years ago that my mother hinted there was something in my past that I didn*'*t know about* … *we never really talked openly to each other*…” ( Daily Mail, 20.03.1998)

Later reports capitalised on stories of single women seeking sperm outside of the clinical context, who were reported to have had unprotected sex with men they had met either online, or in a nightclub.

*Lara*, *from Penryn*, *Cornwall*, *heads to nearby Falmouth to snare her male victims*. *She knows she is playing a lottery with her health*… ( The Sun, 17.08.2010)

The inflammatory language used to describe women using ‘informal’ donation arrangements – as ‘*so desperate for a baby I grab strangers for sex on nights when I*'*m most fertile*’ (The Sun, 17.08.2010), and ‘*so desperate for a baby*, *she resorted to a quick fumble with the plumber*’ (The Sunday Times, 05.10.2008) – was particularly prevalent in, but not exclusive to, tabloid reports. Moreover, although personal narratives were not generally employed in the news in 1988–1992, reports at that time also discussed such ‘informal’ arrangements in negative terms, and in all three time periods, it was suggested that women pursuing parenthood in this way may be putting their sexual health at risk. These articles made frequent references to the contraction of the AIDS virus, echoing research on the role of the disease in ‘spoiling’ social identities (Joffe, 1995).

### Traditional/non‐traditional

Also present across all three time periods was an emphasis on single women using donor sperm as having deviated from ‘traditional’ reproduction with a male partner. Reports often either referred to or directly quoted mothers who described their decision as ‘Plan B’: that is, not what they would have chosen, had the ‘traditional’ family been an option.

*She never doubted that one day she would have her own children*, *as a result of a happy*, *stable relationship*. *But just in case*, *she began to think of alternative ways to motherhood*. *Charlotte was 26 when plan B was devised*. *Six years later*, *the ideal father had still not presented himself*, *so she put her plan into action*. ( The Independent, 14.03.1991)

*Karen herself*, *who runs an exhibition management agency from her home in Twickenham*, *Surrey*, *admits that her position is not ideal*, *but argues that she made the best choice she could given her circumstances*. ( Daily Mail, 31.10.2009)

In most of these articles, single women's use of donor sperm was described as resulting from the ‘choice’ to have a ‘traditional’ family having been taken away – by a failed relationship, a partner who did not want children, the ‘biological clock’ ticking, or another external factor. The ‘traditional’ family concept also permeated articles that emphasised the relationship between single women and the donors involved in conception. In some cases, this relationship was presented as potentially or ultimately romantic. This was particularly the case in articles from 2008–2012, and especially present in articles about websites that match intending parents to potential donors.

*Carol found prince charming in a sperm bank*: “*He*'*s stellar in the sciences*… *He*'*s tall and slim – with dimples*! *And even*, *straight teeth without braces*! *He*'*s a great addition to the family tree*.” *When Jenny first saw a picture of her sperm donor on the web she thought*: “*Oh my god*, *I would totally have sex with that guy*.” ( The Times, 03.01.2008)

Conversely, in 1988–1992, reports capitalised on the fact that single women using donor sperm were rupturing the relationship between sex and reproduction. Specifically, the term ‘virgin birth’ was frequently used in both broadsheet and tabloid news to describe single women seeking fertility treatment. As in reports about lesbian mothers (Alldred, 1998), several articles referred to the views of ‘expert’ individuals and groups – particularly Conservative MPs and religious leaders – reportedly opposed to ‘virgin births’. These arguments mostly focussed on the inability of single women seeking sperm donation to form romantic relationships, their inability to parent effectively, and the resultant negative outcomes for the children born into their ‘non‐traditional’ families. Specific concerns were raised about mothers' sexuality (or lack thereof) and the impact this may have on children's development. In fact, uses of the ‘traditional’ family concept were most pronounced in articles that raised concerns about children's welfare. In 1988–1992, many reports mentioned children's ‘need for a father’, either by quoting religious leaders or Conservative MPs, or in presenting the view of journalists themselves. In 1998–2002, reports were also likely to suggest that children without knowledge of the identity of their biological father would be negatively impacted.

*Society expresses its concern by obliging the doctor to do what virtually everybody would see as the duty of any partner in conception*: *to have regard for the welfare of the child‐to‐be*. *Most would accept that the presence or absence of a father should at least be a factor in assessing that welfare*. ( The Times, 07.05.1991)

*As the law stands in the UK*, *children born* … *through a clinic will only ever know one half of their family tree*. *How are these children going to feel when they discover that their mother has made a deliberate decision precluding any possibility of completing the jigsaw puzzle*? ( The Guardian, 10.06.2000)

Emphases on these ‘missing links’ were common in articles about single women using fertility treatment in, or donor gametes from, other countries, in the so‐called ‘*global fertility bazaar*’ (The Guardian, 30.07.2008). As in media representations of older motherhood (Campbell, 2011), poverty (Chauhan & Foster, 2014), global warming (Smith & Joffe, 2012) and epidemic infectious diseases (Washer, 2004), some articles distinguished between UK services and health services offered elsewhere. Although some reports in both 1998–2002 and 2008–2012 described the ‘traditional’ genetic links formed between children whose mothers used the same sperm donor, others focussed on the possibility of incest between so‐called ‘donor siblings’, and others still described specific cases outside of the UK in which genetic siblings had used fertility treatment together.

### Natural/unnatural

In keeping with the presentation of single women seeking donor sperm as responding to the inviolable link between womanhood and motherhood, articles in all three time periods frequently depicted the decision to use sperm donation as resulting from the ‘natural’ desire to become a mother, albeit in highly ‘unnatural’ circumstances.

“*I*'*m not doing this from choice – but out of desperation*,” *admits Kelly*, *41*. “*I*'*ve never imagined life without a child*.” ( Daily Mirror, 06.01.1998)

“*Being a single mother wasn*'*t what I wanted*, *or hoped for*, *or dreamt of*. *But not being a mother was unendurable*.” ( Daily Mail, 18.02.2010)

Several articles also suggested that mothers were motivated by ‘desperation’ and ‘obsession’, and, in some reports, the desire to parent alone was depicted as an example of ‘nature’ having gone too far, a discourse also apparent in media reporting on late‐age pregnancy (Léchot & Glăveanu, 2013). In the present study, this was particularly true of reports about ‘Octomom’, which frequently described Suleman's ‘obsession’ with babies, and her unashamed determination to have more children despite her lack of employment, and the size of her existing family. Here, the ‘natural’ need to have a child was presented in both tabloid and broadsheet press as pathological, and the language around this presentation – as in the “*tawdry nightmare* [of] *Suleman and* … [her] *repugnant freak show*” (Daily Mail, 02.02.2009) – was particularly striking. At the same time, and as found in other research (Peterson, 2014), the alternative of remaining childless was mostly written out of the news, or was otherwise portrayed as undesirable.

Many articles also presented single women using donor sperm as explicitly and unquestioningly ‘unnatural’. In 1988–1992, ‘virgins’ seeking sperm donation were described as ‘*going against the natural way of life*’ (The Independent, 12.03.1991) and having ‘*a child in* [a] *highly unnatural way*’ (The Times, 12.03.1991). Later articles focussed on scientific developments that may lead to the creation of ‘artificial sperm’, reported to be ‘*tampering with nature one step too far*’ (The Sun, 11.07.2001) and problematically producing ‘*mothers without men*’ (The Guardian, 10.06.2000). In fact, the possibility that men may be written out of the reproductive script featured heavily in reports across all three time periods. Suggestions of the ‘natural’ need for a father were also marked in both tabloid and broadsheet press, and the role of fathers in satisfying children's ‘natural’ need to know their origins was frequently mentioned, echoing arguments found in research on public attitudes to ARTs (Walker et al., 2007).

*Eight years on*, *Viki says she is no more concerned for clues to Alex*'*s paternity than she was when he was born*… *But does she not think a child has the right to know who his natural parents are*? ( Daily Mail, 02.03.1999)

*Josephine Quintavalle*, *of the Comment on Reproductive Ethics group*, *said*: “…*Nature itself teaches us that* … *children are meant to have both a mother and a father*. *When we tamper with the natural order*, *children will always suffer as a result*.” ( Daily Mail, 16.07.2009)

A significant number of articles also suggested that the number of single women seeking donor sperm was rising, and that it would continue to do so. In articles published during 2008–2012 in particular, this increase was described in highly negative terms, and reports often emphasised the age at which women opted to use fertility treatment as problematic (that is, when either ‘too young’ or ‘too old’). It was typical for headlines to capitalise on this aspect, in reports such as ‘*So desperate to be a mum I used a sperm donor* … *at 27*’ (Daily Mirror, 05.04.2011), ‘*At 66*, *is this woman too old to have a baby*?’ (The Sunday Telegraph, 17.05.2009), and ‘*This woman of 72 spent* £*30*,*000 on six courses of IVF*. *And she*'*s still trying for a baby*’ (Daily Mail, 14.07.2009). In fact, articles critical of the number of single women using donor sperm often described multiple features of mothers' identities, and most often, those at either extreme of the spectrum, whether relating to their sexuality (either seeking one‐night‐stands or using donor sperm as a ‘virgin’), their career (either high‐flying or unemployed) or their appearance (either well turned‐out or unattractive).

The presentation of single motherhood by sperm donation as ‘unnatural’ was also striking in reports that depicted the growing cohort as part of a concerning consumerist attitude to parenthood. This was the case throughout all three time periods, with one article including a quotation from a clinician who commented that ‘*if you want a can of beans you can buy one at the shop*, *and some people think the same of babies*’ (The Times, 11.03.1991), and another reporting that the use of ARTs ‘…*is reducing the sanctity of human life to the level of the supermarket trolley*’ (Daily Mail, 20.03.1998). A third, from ‘The Daily Telegraph’ (17.07.2009), detailed that ‘*we have created a conveyor belt of lifestyle choices that defy the laws of nature* … *All you have to do is see what you fancy*, *grab what you want and pay for it*. *No questions asked*.’ A number of reports in both 1998–2002 and 2008–2012 also focussed upon the particularities of consumption, such as women choosing a sperm donor on the basis of specific criteria, or going overseas for treatment, where it was suggested that other women acting as egg donors or surrogates may have been exploited. As key players in the ‘*big business* [of] *donor insemination*’ (The Sunday Times, 31.10.2010), single women seeking sperm donation were also routinely reported to have spent excessive amounts on unsuccessful treatment, and therefore having failed to achieve the ‘natural’ milestone of motherhood.

## Discussion

As in previous research (Correia & Broderick, 2009; Michelle, 2007), the findings of this study demonstrate that single mothers by sperm donation are consistently represented in the British press as ostensibly ordinary, yet ultimately deviant. This representation is present in both broadsheets and tabloids across the political spectrum, and has been maintained in the UK news throughout the years 1988–2012. Findings also highlight the role of three themata in the genesis – and maintenance – of negative portrayals of single women using donor sperm. As rhetorical resources explicitly drawn upon in mass mediated communication, the themata identified as central to this representation are clearly related to ideas about what is ‘traditional’ and ‘natural’ in family life, and rest upon familiar, ‘human’ categories. Building upon Marková's (2003) ideas, these themata appear to be examples of those ‘basic’ or essential to human existence, an argument that goes some way to explaining why representations of family life have been described as ‘structured according to representations of an ‘archaic’ and ‘organic’ character’ (Wagner, 1998:320). If such representations are generated by themata that are fundamental and hence perhaps resistant to challenge or change, it seems unsurprising that representations of new forms of family building are negative in nature. This theoretical insight also provides an explanation for the finding that representations of single women using donor sperm remain consistent over time. Although results make clear that the content of news coverage on this topic is subject to surface changes (from ‘virgin births’ to ‘Octomom’), it is nonetheless evident that the representation of single women using donor sperm as ‘other’ retained its potency in the British press from 1988–2012.

Why might this finding be of concern to social psychologists who have a commitment to social justice? One answer is found in Walker et al.'s (2007:173) suggestion that anchoring arguments about ARTs in ‘archaic’ structures of meaning enables evasion of the ‘opprobrium of being unjust and discriminatory’. With regard to single mothers by sperm donation, it is clear that media representations bear little relation to the psychological literature that has shown that this user group – and their children – are psychologically well‐adjusted, and their families are characterised by positive mother–child relationships (Golombok, Zadeh, Imrie, Smith, & Freeman, 2016). It is also noteworthy that such representations proliferate despite regulatory changes that now explicitly permit the use of donor sperm by single women (Human Fertilisation and Embryology Act, 2008). Given the role of mass mediated communication in producing and propagating ideas in modern societies, this finding is clearly problematic. While conclusions cannot be drawn from the present study about the impact of media representations on public perceptions, it is worth reiterating that the relationship between media representations and public understandings of ARTs may be more marked than it is for other phenomena, which either may not relate to ‘basic’ themata, or about which the general public may have access to other sources of information.

Findings also illustrate that media representations of single mothers by sperm donation increasingly rest upon women's own narratives of their experiences. Although part of a wider trend in both print and online press (Turner, 2010), the use of this device in relation to this group is noteworthy, given that single women using donor sperm are consistently reported to have described themselves as having no choice but to deviate from the ‘traditional’ family and ‘natural’ conception, and as being concerned with ‘key’ issues such as fatherhood and genetic connections. On some level, these narratives may be said to correlate with some of those found in scholarly research (Zadeh, Freeman, & Golombok, 2013). However, what is consistently lacking in the media on this topic are reports that include the questions asked of mothers in order to produce these accounts, and/or the accounts of mothers who do not depict their experiences in these terms. Although generally portrayed as spontaneous reflections on motherhood, from an SRT perspective, the use of personal narratives is of concern insofar as they relate to Jovchelovitch's (1997) argument that the public sphere is characterised by ‘unequally equipped agents’. Seen in this way, it is clear that the propagation of particular personal experiences in the news equips specific ‘local knowledges’ with resources that (i) would not otherwise be available, (ii) may reflect some ‘local knowledges’, and not others, and (iii) may not reflect such ‘local knowledges’ accurately (if at all). Beyond Michelle's (2007) argument that media professionals may deliberately include the narratives of particular people that ‘fit’ with a predetermined news angle, the findings of this study therefore incite consideration of how and why such narratives are produced as they are, by whom, for what purpose, and with what effects.

Moreover, unlike other research that has demonstrated that personal narratives may be used in order to generate a sympathetic perspective towards users of mental health services (Foster, 2006), in the case of single women using donor sperm, this rhetorical device arguably does the exact opposite. One possible explanation for this difference may be that mediated representations of users of mental health services and sperm donation are respectively and differentially generated through a process of ‘emotional anchoring’ (Höijer, 2010). While in the former case, the user perspective is seemingly deployed in order to reduce the fear that generally underpins public understandings of mental ill health, in the latter, narratives may reflect the fact that representations of single mothers by sperm donation are anchored against a backdrop of more familiar forms of family building (Wagner, 1998), and therefore in fact *attached to* emotions of fear. Findings are therefore relevant to theorising the relationship between ‘basic’ themata and ‘emotional anchoring’ to ‘archaic’ structures of meaning. Specifically, the idea that ‘basic’ themata ignite a process of ‘emotional anchoring’ might explain firstly the social legitimacy afforded to social representations that discriminate against particular groups, and secondly, the resistance of these representations to change in the face of new and/or conflicting information. These two lines of argument are important for thinking about the efficacy of social interventions designed to address such representations, and it is noteworthy that the previous literature on public understandings has also determined the role of personal testimonies in the generation of social representations that are anchored in – although in some cases dispute – the view that the ‘traditional’ family is optimal (Walker et al., 2007; Correia & Broderick, 2009).

In contrast to the finding that there are different ideas held by different groups regarding the use of ARTs by single women, the present study found no differences in the overall presentation of this user group in different media sources. However, differences were identified in the number of articles published on the topic, with right‐leaning newspapers drastically augmenting their coverage over time. This increase might be understood as something of a ‘kick back’ against the removal of references to children's ‘need for a father’ in legislation that regulates professional decision‐making about fertility treatment provision (Human Fertilisation and Embryology Act, 2008). Findings therefore incite theoretical reflection on the relationship between the three dimensions of the public sphere identified by Gaskell and Bauer (2001). According to this model, attention should be paid to the relationship(s) between representations in three arenas: mass mediation, public perception and political regulation. In the case of social representations of single mothers by sperm donation, it seems that the relationship between mass mediation and political regulation, with regard to right‐leaning media at least, is best characterised as antagonistic. In addition, the fact that increases in media coverage were not identified across all sources suggests that there may be benefits to drawing within‐group distinctions in conceptualisations of mass mediated communication, and to seeing the media as a conglomeration of different groups (Bauer & Gaskell, 2008), who, with their intended audiences, and against a particular political backdrop, each contribute to the production, distribution and reception of representations of single women using donor sperm in different ways. In this way, findings invite further engagement with Moscovici's (2008) early insights on different communicative genres as they apply to different media sources.

That different media unequally attend to this issue over time is also noteworthy given that the media has been said to play a unique role in representational construction (Wagner, 1998) by its capacity to depict specific social issues as publicly salient (Gaskell & Bauer, 2001). However, the relationship between mass mediation and public perception is not linear, and it is worth considering that different media sources may produce and disseminate different amounts of ‘information’ about single mothers by sperm donation in anticipation of reader responses to increasingly permissive legislation. In part, this may explain why the media rely upon personal narratives (with intended emotional appeal). Findings therefore necessitate renewed emphasis on the fact that the relationship between different dimensions of the public sphere may change over time (Bauer & Gaskell, 1999) in ways that have implications for what is produced – and what proliferates – in each component. Such insights incite further consideration of the ways in which knowledges produced either by the media or in the political arena are deemed legitimate or illegitimate (Jovchelovitch, 1997), both by each other, and by the general public.

Despite previous theoretical work that has suggested that new technologies become normalised in mass mediated communication over time (Wagner et al., 2002), findings here suggest that ARTs may constitute a specific category of biotechnological innovation that is neither initially abnormalised nor ultimately normalised within the British press. It is possible that the continued ‘othering’ of single mothers by sperm donation in the UK press is connected to continued regulatory changes related to this group. A second possibility is that there exists a distinction between media representations of biotechnologies in and of themselves and media representations of the *users* of such technologies in particular. Whether or not similar findings would have been generated had the focus of this study been on media representations of the ‘technology’ of sperm donation – rather than on one of its user groups – is not known. It is also not clear that similar findings would have emerged from an analysis of both textual and visual media content relating to this topic. Indeed, the use of electronic, textual archives as the primary method of data collection suggests that findings ought to be considered with caution.

Although not without its limitations, the present study provides a useful insight into UK media representations of the users of ARTs in general, and single mothers by sperm donation in particular. Given the suggestion that media reports of ARTs may be depended upon by the general public, and previous findings that media‐reported personal narratives may be more persuasive of readers than reports of a more factual nature (Hargreaves, Lewis, & Speers, 2003; Provencher, 2007), the implications of this study are considerable. It is perhaps not coincidental that previous studies of professional and public attitudes towards single women using donor sperm have established that this group are generally met with social disapproval. Further research on the psychosocial impact of media representations of single mothers by sperm donation is now recommended.
